# On aims and methods of psychiatry – a reminiscence of 50 years of Tinbergen’s famous questions about the biology of behavior

**DOI:** 10.1186/s12888-014-0364-y

**Published:** 2014-12-21

**Authors:** Martin Brüne

**Affiliations:** Division of Cognitive Neuropsychiatry and Psychiatric Preventive Medicine, LWL University Hospital, Ruhr-University Bochum, Alexandrinenstr. 1, 44791 Bochum, Germany

**Keywords:** Evolution, Mechanism, Ontogeny, Phylogeny, Adaptation, Psychiatric conditions, Emotions, Mismatch, Dysfunction, Psychotherapy

## Abstract

**Background:**

In 1963, Nicolaas Tinbergen published an article on “the aims and methods of ethology” in which he identified a fundamental framework for the scientific inquiry into the understanding of biological phenomena. In particular, he emphasized to not only study what he called the “proximate” causes, that is, mechanism and ontogeny of a given trait, but to include evolutionary explanations, i.e., the phylogeny and adaptive properties of that trait.

**Discussion:**

While influential in the field of biology and to some degree medicine, psychiatry has fallen short of adopting Tinbergen’s approach. This article aims at discussing why Tinbergen’s précis has lost nothing of its attractiveness to psychiatry as a medical discipline. Examples will be given for the analysis of emotions, attachment and psychotherapy.

**Summary:**

Tinbergen has bequeathed to us a scientific framework that can greatly advance our understanding of psychiatric conditions and improve diagnosis and treatment of mental disorders, with similar potential for medicine in general.

## Background

Fifty years ago, in 1963, Nicolaas Tinbergen, at that time Lecturer at Oxford University, United Kingdom, published a paper entitled “*On the Aims and Methods of Ethology*” on the occasion of Konrad Lorenz’s 60th birthday. Both Tinbergen and Lorenz, together with Karl von Frisch, were awarded the Nobel prize in medicine in 1973 for their ground-breaking work in the field of ethology). Tinbergen’s paper became influential in biology, because it provided a précis of scientific inquiry into the biological study of (animal) behavior. In particular, Tinbergen pointed out that, for a full understanding of an observable biological phenomenon (movement or behavior), it is necessary to analyze four different, though related, aspects: its causation (or mechanism), its ontogeny (developmental trajectory), its survival value (biological fitness) and its evolutionary (phylogenetic) history. Scientific inquiries into mechanisms and development are referred to as the “proximate” causes [1], because they are malleable during an individual’s lifetime and strongly depend on gene-environment interaction, and, as we know today, epigenetic mechanisms [2]. The other two aspects, fitness and phylogeny have been called “ultimate” or evolutionary causes. Understanding the evolutionary causes of behavior entails exploring similarities and dissimilarities between phylogenetically related species (comparative method), and the analysis of the ecological contingencies in which a given trait was shaped by selection [1]. Each area has a corresponding question about the trait: How does it work? How does it develop? What is its phylogeny? How has it influenced biological fitness, that is, survival and reproductive success?

Tinbergen’s article shortly followed Ernst Mayr’s efforts to distinguish “proximate” from “ultimate” explanations. Mayr’s book, *The Growth of Biological Thought*, describes the history of biology as intertwined strands that nonetheless remain remarkably separate, one about mechanisms and their ontogeny (proximate), the other about why traits are the way they are, that is, evolution [3,4]. “Evolutionary” has mostly replaced the term “ultimate” to avoid its slightly supernatural tinge [5]. Mayr notes, “No biological problem is solved until both the proximate and the evolutionary causation has been elucidated. Furthermore, the study of evolutionary causes is as legitimate a part of biology as is the study of the usually physico-chemical proximate causes.”

Why is this relevant to psychiatry? Psychiatric research and neuroscience has made tremendous progress in exploring the proximate causes of psychopathology, that is, its underlying mechanisms (e.g., aberrant neurotransmission, abnormalities in brain structure and function, discovery of genes increasing the risk for dysfunction etc.). By comparison, relatively few studies have explicitly sought to address psychiatric conditions by taking into account the adaptive properties of the brain/mind including behavior, cognitive traits or emotions etc.. Indeed, such an approach is all but straightforward, it seems, because many clinicians would intuitively argue, from a medical point of view, that bodies are optimal by evolutionary design, suggesting that every (behavioral) dysfunction is an expression of a pathological process affecting the respective output organ (e.g., the heart or the brain). Related to this, dysfunction is often deemed qualitatively different from function, which remains in the most recent classification of psychiatric conditions, the DSM-5 [6]. This view, however, disregards observations that most, if not all, psychopathological signs and symptoms differ from “normalcy” be degree, not kind.

In this article, it is proposed that human behavior – both normal and pathological – can be, and needs to be, studied along the lines proposed by Tinbergen, which decisively includes an evolutionary perspective. Specifically, it is asserted that psychiatry (and medicine in general) cannot make progress without acknowledging the fact that human cognition, emotions and behavior have evolved, and continue to evolve [7], in precisely the same way as any other observable phenomenon in any living creature. Without a deeper understanding of the biological and environmental contingencies, including their interactions, and of the evolutionary history of any given traits, many psychopathological conditions remain elusive with respect to their origins, which limits potential treatment options.

This article takes the 50th anniversary of Tinbergen’s publication as an opportunity to consider how his four questions can clarify the aims and methods of psychiatry. It is contended that a systematic application of Tinbergen’s questions can help psychiatry the way they have advanced the study of animal behavior. A few examples will be offered to illustrate the opportunity; emotions and their disorders, attachment and its disorders, and some potential implications for treatment. A question central to evolutionary medicine is at the core of this approach: Why has natural selection left the human mind so vulnerable to dysfunction? Related to this, one can ask more specifically: Why do negative emotions persist, given the suffering they cause and their role in causing disease? Why do so many people have attachment patterns that are insecure, given the dire effects on relationships? How can all this help to tailor individualized psychotherapy?

### Natural defenses, “smoke detectors” and environmental mismatch

Homo sapiens is a product of evolution by natural selection. Although many characteristics evolved in our ancestral past as hunter-gatherers, known as the “environment of evolutionary adaptedness” (EEA; [8], there is evidence to suggest that our species continues to evolve and to adapt to new environmental challenges [7]. In the evolutionary past, humans faced threats in many divergent domains, ranging from microbial pathogens attacking the immune system to inter-group conflict between competing tribes. Thus, the EEA was anything but a stable condition; in fact, as environmental contingencies changed, so did adaptive solutions. Put differently, evolution by natural selection has continuously produced answers to changing biological challenges affecting reproductive fitness, whereby many different defense mechanisms emerged, often following the “Red Queen” principle (so named after “Alice in Wonderland” whereby the Red Queen runs ever faster just to remain where she is!) or in an “arms race” fashion [9]. This proposes that organisms must constantly adapt, evolve, and proliferate not merely to gain reproductive advantage, but also simply to survive while pitted against ever-evolving opposing organisms in an ever-changing environment. Viewed this way, it follows that bodies, organs or individual traits are never optimal by design, but work sufficiently reliably to maintain homeostasis and to respond to imbalance thereof. With regard to defense mechanism, the hypothalamic-pituitary-adrenal system has properties that enable organisms to cope with stress, yet there are huge between-species differences with regard to the regulation of chronic stress [10] and within-species differences with regard to stress resilience [11]. A substantial number of people now suffer from posttraumatic stress disorder (PTSD) that is often precipitated by adverse events such as neglect, abuse or other severe psychological trauma [12]. Such traumatizing events were perhaps not prevalent enough in our evolutionary past to impact biological fitness. Hence, no specialized or unique coping mechanisms evolved to these “unnatural” challenges [13].

Fear responses to real or imagined environmental threats may be elicited by contextual factors that more or less resemble ancestral hazards. Insight comes from signal detection theory suggesting that smoke detectors operate at low thresholds, which comes at the risk of producing false alarms. Smoke detectors are purposefully designed so by human technicians, because a missed alarm response would be more costly than a false alarm. Nature has selected defense responses that work analogously, in principle, to a domestic smoke alarm or detector. For example, anxiety may help protect an individual from falling prey to a predator or succumb in intra-species conflict [14]. Likewise, low mood or (mild) depression may occur as a protective response to social stress, wherein an individual feels entrapped in unresolved conflict or defeated in social contest [15]. Depression and anxiety can thus be considered in psychiatry as analogous to cough and fever in medicine. They are undesirable, but often necessary defenses against environmental adversity, and may help re-implement physical and psychological homeostasis [16]. Such mechanisms may themselves become disadvantageous or even life-threatening, if they progress beyond a certain critical value or “threshold”. Like fever and cough can promote sickness and even death (if unsuccessful in eliminating their pathogenic cause), anxiety and depression can become highly dysregulated (e.g., if defeat or entrapment become chronic), such that individuals are no longer able to cope with the situation and instead, may develop suicidal ideas [17]. Similar responses to threat can be observed in nonhuman primates and other animals, and abundant research in this area has elucidated the underlying defense mechanisms e.g., [18-20]. These examples, presented in a nutshell, may illustrate the importance of inquiry into the evolutionary causes of emotions, that is, their phylogeny and adaptive properties.

When reading Tinbergen’s paper carefully, one recognizes that the “three major problems of biology” (mechanism, phylogeny, adaptive value) were already carved out by Julian Huxley [21] to whom Tinbergen gives full credit for his insights [1, p. 411]. The fourth dimension of scientific inquiry, ontogeny, was added by Tinbergen. It is perhaps the most relevant for the understanding of human behavior and psychopathology, because it refers to the pertinent question of “innate” versus “acquired”, “instinctive” versus “learnt”, or “nature” versus “nurture”. In fact, as we will see in the next section, it was one of Tinbergen’s great insights to discover, and lay down the theoretical foundation of, the “ontogenetic interaction between the individual and its environment – which, incidentally, takes the form of trial and error only where evolution has not given precise direction to the ontogenetic process” [p. 426] – that is, an individual’s behavioral ecology.

### Attachment, life history patterns, and reproduction

Mammals, and to a lesser degree birds, represent a taxonomic group of animals that critically depend on parental care during the early stages of development [22]. In the primate lineage that led to the evolution of Homo sapiens, the extremely prolonged periods of infancy and childhood fostered the emergence of empathic concern for others [23]. One decisive ecological factor that accelerated the evolution of close relationships between infants and caregivers in human predecessors was a change in climatic conditions with the emergence of open savannas, which provided selection pressures favouring bipedalism (upright walking). Over evolutionary time, the brains of ancestral bipedal hominids started to grow larger due to other selection pressures related to increasing social complexity [24]. Bipedalism, combined with the limits of the female pelvis with and the requirements for giving birth to infants with large brains, necessitated the trade-off of giving birth to increasingly immature offspring.

This prime example of an evolutionary design compromise is perhaps the one with the furthest-reaching consequences for mental life. Indeed, “the obstetrical dilemma” [25] has profoundly impacted human parenting, mating behavior, as well as the formation of trustful and supportive relationships [26]. Immaturity of human newborns, for instance, favored the selection of close dyadic relationships between mother and offspring. The British psychoanalyst John Bowlby was probably the first to recognize the importance of early mother-child relationships for mental health. He coined the term “attachment” [8] for proximity-seeking behavior of the baby, which, according to Bowlby, was “preprogrammed” (instinctual) as to secure survival. Interestingly, when Bowlby started to present his ground-breaking ideas, he was almost expelled from his psychoanalyst community, because he apparently insisted on the relevance of (early) human interaction for mental health, rather than acknowledging the primacy of intrapsychic conflict as key to psychopathology [27]. Consequently, Bowlby sought help in the camp of ethologists, foremost Robert Hinde, who were much more open to his ideas, as experimental research in nonhuman primates readily substantiated Bowlby’s insights e.g., [28].

Subsequent research in humans revealed that infants differ with regard to their attachment style. The majority develops a so-called secure attachment style, which is characterized by the development of trustful “inner working models” (IWM; [8]. Trustful IWMs develop when caregivers are responsive to infants’ needs. That is, caregivers (not necessarily the biological mother) recognize signs of distress in the child, are emotionally available, provide a “safe haven” and validate children’s emotions in supportive ways. Through this interaction, the child learns to appreciate the world as a safe place, which forms the basis for exploring the environment and becoming independent over time. This includes the ability to form coherent internal images of self and others as individuals with distinctive mental operations.

Insecure attachment, in contrast, is more likely to develop when caregivers are less or even unresponsive to their infants’ needs. In fact, harsh and rejecting parenting styles lead the child to appreciate the world as an unsafe place, others as unreliable and untrustworthy.

Based on how young children respond to a “strange situation” [29] in which the child is first separated from and subsequently reunites with her caregiver, attachment theorists distinguish between anxious-ambivalent (clinging), avoidant-ambivalent (pseudo-autonomous) and disorganized attachment (freezing, stereotypic behavior).

Bowlby believed that secure attachment was the only adaptive attachment style, and the others deviants from the norm. This view, however, somehow disregarded the ecological contingencies on the side of the parents (or caregivers).

More recent conceptualizations of attachment suggest that it could indeed be adaptive to accelerate the reproductive cycle, when resources are scarce. Hence, if environmental conditions are unstable or unpredictable, caregivers may actually invest less in offspring compared to conditions where resources are abundant. Viewed this way, insensitive parental rearing styles not only promote the development of mistrustful inner working models, they also lead the offspring to reproduce earlier as prospects of future resource availability are gloomy [30]. In such a situation it might be biologically adaptive to be less discriminative with regard to mate choice, to be exploitative in terms of interpersonal relationships, and invest little in own offspring, which includes limited emotional availability.

Early environmental contingencies not only shape one’s psychological development. There is now convincing evidence for acceleration or delay of biological maturation, depending on early developmental circumstances [31]. For example, girls who grow up in families in which fathers are absent or familial conditions are otherwise stressful, reach menarche earlier compared to families with both parents being present [32]. That is, children who grow up in families with spousal harmony, low levels of stress and adequate resources may also be experience greater emotional availability of their caregivers (thus more often develop secure attachment), which leads to later biological maturation and mating. Also, securely attached individuals seem to have more stable long-term intimate relationships compared to individuals who as children have experienced greater adversity. From a psychopathological point of view, it is clear that differences in life history patterns do not necessarily correspond to mental health. Rather, it is obvious that evolution has not selected for well-being, but instead, for maximizing reproductive success. Consistent with this notion, individuals who grew up under adverse conditions are more likely to show more signs of psychological distress, with females presenting more internalizing problems (such as anxiety and depression), whereas males more often display externalizing problems (antisocial behavior, addiction). In extension to this, the tendency to choose a mate with a similar psychological profile (assortative mating) may aggravate the situation. For example, depressed women seem to often choose men as partners with antisocial personalities, which increases (non-genetically) the risk of offspring for developing a psychiatric condition [33].

Even though individual differences in behavioral ecology seem to be mainly environmentally driven, there is evidence to suggest that attachment style is influenced by genetics [34]. However, the role of genetic variation cannot be meaningfully interpreted without taking into consideration the interaction of alleles with environmental contingencies.

In the context of gene-environment-interaction it seems necessary to reframe the classic “diathesis-stress-model”, according to which individuals are rendered more vulnerable than others to developing psychological problems depending on their genetic make-up. For example, research has shown that individuals carrying the “short” variant of the serotonin transporter coding gene predisposes to depression in individuals who have experienced early adversity such as neglect or abuse [35]. Similarly, carriers of the low-activity variant of the MAO-A coding gene are at greater risk of developing antisocial personality disorder when growing up under adverse conditions [36]. Diathesis-stress models are, however, insufficient to explain the following observations: One is that several of the “vulnerability” genes have been positively selected during human evolution. From an adaptationist point of view, it is simply implausible to assume that natural selection has favored an increase in frequency of allelic variants that convey vulnerability to psychological dysfunction. Second, there is growing evidence to suggest that the very same genetic variants that only increase the risk of developing a psychiatric condition if associated with adverse rearing conditions, can lead to better than average outcomes when meeting favorable early environmental condition (high-quality care-giving). In support of this discovery, labeled “differential genetic susceptibility”, research has revealed that the low-activity variant of the MAO-A coding gene is associated with *lower than average* prevalence of antisocial personality when individuals grew up in supportive environments [37]. Along the same lines, carriers of the s-allele of the 5-HTTLPR have a *lower risk* for depression under beneficial early developmental conditions [38]. Moreover, individuals carrying the seven-repeat variant of the DRD4 gene may be exposed to a *greater than average* risk for ADHD and externalizing problems, if growing up under unresponsive care, whereas their risk for ADHD is *lower than average*, if their mothers’ responsivity is superior [39]. Taken together, early environmental conditions have a decisive impact on how people perceive the world, form social relationships, and even mature biologically. Selection has endowed humans with a set of highly flexible responses to different contingencies that evolved to maximize reproductive success. Such “open programs” [40] have the potential to produce both emotionally stable and unstable personalities, but neither was a direct “goal” of evolution. In light of profound skepticism of clinicians that evolutionary insights can contribute to improving mental health [41], it is now crucial to demonstrate exactly this.

### Evolutionarily informed psychotherapy

The general purpose of psychotherapy is to improve patients’ subjective well-being and reduce suffering by means of verbalizing interpersonal problems, validating patients’ needs, and change attitudes, emotional appraisal of difficulties, and problem behavior in the context of a trustful client-therapist relationship. Studies have shown that no one therapeutic “school” is superior in efficacy over another. Instead, it seems that the quality of the therapeutic relationship is most essential [42]. This is unsurprising, given the relevance of early social interaction for the formation of attachment and the pervasiveness and transference of attachment styles to other relationships such as those to peers, friends, colleagues, romantic partners and own offspring [27].

In an ideal world, primary prevention would aim at aiding parents to provide optimal emotional care and resources to their children, which could help infants to develop secure attachment. In fact, preventative interventions in problem families has revealed remarkable effects on child development [43]. This may, however, be an unrealistic goal for all to attain, yet therapeutic nihilism is unwarranted in light of evidence for the malleability of attachment styles over time [44]. Indicated prevention (which, by definition, concerns the identification of individuals who are experiencing early signs or symptoms, but have not yet reached the point where a clinical diagnosis can be made) may be relevant for those individuals who carry a substantial number of genetic variants that are associated with increased differential susceptibility, since research has shown additive effects with regard to an individual’s responsivity to early environmental conditions, mainly emotional availability of caregivers [45]; though it is premature to advocate genetic testing and drawing simplistic therapeutic or predictive correlations.

In addition to these insights from attachment theory, the relevance of the evolutionary perspective assists updating and reinterpretation of some classic psychoanalytic claims. One refers to the debate about incest taboo and Freud’s seduction theory, according to which infant sexuality fosters the oedipal conflict. There is now evidence to suggest that psychoanalytic theory, in part, confused cause and effect. Rather than proposing an unconscious wish of young children to have sex with the opposite-sex parent, it is plausible to assume that several of Freud’s patients were actually victims of sexual abuse in the family, suggesting that many symptoms that Freud observed emerged from early trauma [46]. In addition, abundant research has shown that nature has selected mechanisms of incest avoidance, which helps prevent the accumulation of deleterious mutations [47].

Although clinicians rarely identify the contribution of evolutionary theory to psychotherapy, Tinbergen’s ultimate questions have also had considerable impact on psychoeducation in the cognitive behavioral treatment of anxiety disorders, which includes models of causality explained to patients akin to the smoke detector principle [48]. More specifically, recent psychotherapeutic developments such as metacognitive therapy, mentalization-based therapy and compassion-focused therapy explicitly refer to the ultimate causation of cognition, emotions and behavior [49–51], with minor differences with regard to their relatedness to classic attachment theory.

Another critical, and widely neglected, aspect pertaining to patient-therapist interaction refers to the analysis of nonverbal behavior during clinical settings. Studies have demonstrated the predictive power of ethological analyses of nonverbal interaction with regard to treatment response and outcome that is superior to the utilization of classical rating scales [52]. Ethological methodology as proposed by Tinbergen [1] assigns nonverbal pattern of behavior a communicative meaning. That is, drawing on between-species as well as cross-cultural comparison, behaviors observed in nonclinical subjects or psychiatric patients, including those during social or therapeutic interaction, are nonverbal correlates of internal emotional or motivational states. For example, crouching postures, averted gaze or self-directed activities such as grooming or locomotion are nonverbal correlates of defense or ambivalence (fight or flight).

Following these lines, numerous studies have demonstrated that patients with psychiatric disorders can be distinguished from nonclinical subjects on the basis of their nonverbal behavior during social interaction [53]. Furthermore, there is convincing evidence that the prediction of therapeutic response is reliably possible at a very early stage of treatment, based on subtle nonverbal signals which include facial movements, body posture and movements directed towards the own body, in ethological language referred to as “displacement activities” [54]; reviewed in [53]. For example, Geerts et al. [55] found that the lack of non-verbal convergence (nonverbal behavior of patient and interviewer becoming more “attuned” over time) between the patients’ amount of verbal material and the interviewers’ encouraging feedback (“yes” nodding, verbal back-channeling etc.) predicted an unfavorable short-term outcome of depression, independent of classic ratings of symptom severity. Most excitingly, the analysis of patients’ and interviewers’ nonverbal interaction has the potential to predict relapse of depression, as was shown in individuals with remitted depression, whereby a reduction in nonverbal convergence predicted relapse within a 2-year follow-up period [56]. These examples clearly suggest that the analysis of nonverbal and paraverbal signals during therapeutic interaction can be more informative than subjective report or information obtained by using standardized rating scales. This probably resides in the fact that nonverbal behavior is less under conscious control compared to verbal communication, such that an individual’s “real” motives cannot so easily be concealed [57,58]. In addition, standard rating scales utilized in clinical assessments usually lump together subjective report and clinical impression. However, although clinicians intuitively use their species-specific endowments for deciphering nonverbal expressions in therapist–client interactions, the extent to which clinical judgments rely on the unconscious perception of patients’ communicative signals is unclear and highly under-researched in clinical psychiatry [59].

## Discussion

Fifty years ago, ethologist Nicolaas Tinbergen provided biology with a framework of the aims and methods of ethology, which, at that time, was the spearhead of contemporary research of behaviour. The appreciation of Tinbergen’s four “Whys”, the proximate and evolutionary mechanisms of behavior, by other behavioral sciences has been mixed. In spite of several attempts to promote the usefulness of Tinbergen’s approach to the understanding of cognition, emotion and behavior of humans e.g., [60], psychiatry has been curiously unaware of the prospects and opportunities inherent to Tinbergen’s ethological methodology for improving the understanding and therapy of psychiatric conditions. This is, in part, understandable, because psychiatrists, with a background in medical education, are trained to see psychiatric phenomena as diseases, as well as pathological deviations from a (unspecified) biological and or social norm. They have much more difficulties in appreciating that some phenomena are better conceptualized as defenses or interpersonal strategies (whereby the term “strategy” does not imply conscious reflection or awareness) that have been shaped by a long history of evolutionary development.

This article proposes that evolutionary approaches contribute important insights into how the human mind has been shaped by selection and how human mentality may be (or not!) adapted to ancient and modern environments. One possible pitfall is to conceive of evolutionary processes as optimal by design. Instead, some features that have been selected at one time may produce vulnerability to dysfunction. For example, low mood or anxiety can be useful in situations, in which conflict is inevitable, or when important biosocial goals become unattainable. Similar to adaptive defenses such as pain, coughing, vomiting, and fatigue, psychological mechanisms such as low mood, withdrawal or fear can help protect the individual from an escalation of conflict and further harm, however, at the expense of subjective wellbeing. Evolution by selection processes ultimately maximized survival and reproduction, not health or wellbeing [61]. This can distort or blind the therapist’s vision with regard to the function of emotions, cognitions and behavior.

Human psychology depends on the complex interplay of genes and early environmental conditions. In particular, the emotional availability of early care-givers coins an individual’s mindsets in terms of one’s ability (or disability) to form trustful and reciprocal relationships with peers, partners, colleagues and additional significant others. Research into the behavioral ecology of humans has demonstrated the enormous flexibility in shaping developmental pathways, including the timing of biological maturation, mating, and the quality of parenting behavior. In spite of potential risks for mental health associated with such differential susceptibility, the fact that there is a large potential of environmental malleability of psychological development – for better and worse – opens new horizons for prevention and treatment of psychiatric conditions. Identifying and promoting favorable early rearing conditions is one such approach in preventing or reducing the vulnerability or liability to developing mental disorders. Moreover, it is more holistic and scientifically complete to consider and help patients understand that problems are not simply related to genetics and adversity (as the classic diathesis-stress approach would suggest), but a complex interaction between genetic variants that may or may not increase one’s responsivity to both good and bad environmental conditions. In this way, patients’ resultant strengths may be identified and optimized. Being endowed with genes that make one more responsive to environmental input could potentially be turned into a therapeutic advantage, that is, greater responsivity to therapeutic intervention.

Another under-used area for clinicians to utilize is the importance of non- and paraverbal signals during patient-therapist interaction. It is clear that therapeutic effort is mainly executed through verbal discourse. However, it would be useful to educate and encourage clinicians to utilize more information from nonverbal behavior, especially in light of claims that roughly 70% of communication during social interaction is transported via nonverbal channels, 25% paraverbally, as opposed to just 5% of information that is verbally expressed [57].

## Summary

To conclude, Tinbergen has bequeathed us a more accurate, informed, holistic and predictive scientific framework that not only advances our understanding of psychiatric conditions, but also improves psychiatrists’ concepts of how a diagnosis should be framed and furthermore suggests proposals for the identification and targeting of treatments of those mental disorders [62,63]. It also has the potential for a considerable impact on medicine in general [5]. It is therefore hugely important to educate all doctors, and psychiatrists in particular, about evolutionary theory, especially Tinbergen’s approach. This should then be reflected in the curricula of medical schools and postgraduate studies, for the benefit of all, especially our patients.
